# Prevalence of Depression, Anxiety, and Post-traumatic Stress Syndrome Among Intensive Care Unit Survivors in Jazan, Saudi Arabia

**DOI:** 10.7759/cureus.60523

**Published:** 2024-05-17

**Authors:** Mohammed Ageel, Abdullah Shbeer, Mariam Tawhari, Hussam Darraj, Maisa Baiti, Raghad Mobaraki, Areej Hakami, Nawaf Bakri, Rahf H Almahdi, Raghd Ageeli, Mawada Mustafa

**Affiliations:** 1 Department of Surgery, College of Medicine, Jazan University, Jazan, SAU

**Keywords:** mental health, anxiety, depression, ptsd, icu survivors

## Abstract

Objective

To quantify the prevalence of depression, anxiety, and post-traumatic stress disorder (PTSD) among ICU survivors in the Jazan region, Saudi Arabia, and explore the correlational relationships among these conditions to inform targeted mental health interventions in this unique regional context.

Methods

The study employed a cross-sectional observational design to assess ICU survivors from two major hospitals in the Jazan Region: Prince Mohammed Bin Nasser Hospital and King Fahad Central Hospital. One hundred participants were interviewed face-to-face to gather detailed insights into their post-ICU experiences. We employed the hospital anxiety and depression scale (HADS) and the post-trauma symptom scale (PTSS-10) to systematically assess the psychological impacts of anxiety, depression, and PTSD among participants.

Results

The demographic breakdown of participants showed a youthful skew, with 37% under 35 years, 49% aged between 36-60 years, and only 14% over 60 years, contrasting with typical ICU demographics, which generally skew older. This younger distribution may influence the psychological outcomes observed. The sample was fairly gender-balanced, with 53% male and 47% female, closely reflecting the regional gender ratio of ICU admissions. Among the participants, 24% were classified as 'abnormal' and 20% as 'borderline abnormal' for anxiety, while 25% were 'borderline abnormal' and 21% 'abnormal' for depression. About 8% of participants were diagnosed with severe PTSD. Anxiety was more strongly correlated with PTSD than depression. The analysis demonstrated significant associations between demographic factors and psychological distress among ICU survivors. Females reported higher anxiety, while lower education and unemployment were associated with increased depression. Additionally, lower household income was associated with higher PTSS scores, and marital status was linked to depression, suggesting that socioeconomic factors play a critical role in post-ICU psychological recovery.

Conclusion

The findings emphasize the imperative need for comprehensive mental health evaluations and tailored interventions for ICU survivors in the Jazan region.

## Introduction

The mental well-being of patients discharged from the ICU has become a cornerstone in contemporary medical and psychological discourse [1,2]. ICU admissions inherently expose patients to acute physical and psychological challenges, from invasive treatments and extended solitude to potential bouts of delirium and the omnipresent risk of mortality [3]. Subsequent to their discharge, survivors often confront a spectrum of psychiatric disorders, including anxiety, depression, and post-traumatic stress disorder (PTSD) [4]. The complex synergy of these mental health issues can substantially hinder their overarching healing journey, casting ripples not just on the affected individuals but also on their familial and social circles [5]. The potentially enduring effects of such mental afflictions amplify the imperative for early intervention and robust support mechanisms. Hence, comprehending and addressing the psychological concerns of ICU survivors stands essential for enhancing patient outcomes and facilitating their smooth transition back to routine life.

Anxiety among ICU survivors has risen as a pivotal issue demanding medical recognition. Their tenure in the ICU, marked by intense medical actions, alien environments, and recurrent physical discomfort, can deeply distress patients. Post-discharge survivors often grapple with harrowing memories of procedures, ventilations, or periods of paralysis [6]. Such memories can evoke severe anxiety symptoms, leading to perpetual apprehension, unease, and invasive recollections of their ICU experiences. The unpredictability of their healing path and potential long-term health ramifications intensify this anxiety. This pronounced anxious state not merely obstructs physical healing but also detracts from their holistic well-being and interpersonal engagement [6]. Therefore, addressing this anxiety remains central to ensuring both physical and psychological recuperation.

Depression among ICU survivors is of paramount clinical and research significance. Their ICU experience, rife with potential mortality, invasive actions, and extended solitude, often triggers feelings of despair and overwhelming sadness. Post-discharge challenges, such as physical constraints, cognitive shifts, and altered self-perception, can compound depressive symptoms [5]. Some might find it arduous to reclaim their prior familial, professional, or societal roles, exacerbating feelings of insignificance. Chronic physical ailments, like fatigue or pain, might further intensify depressive inclinations [1,4]. It's crucial to recognize and address this depression, given its potential to stymie physical rehabilitation, prolong the resumption of daily tasks, and compromise adherence to clinical recommendations.

PTSD among ICU survivors underlines the depth of the psychological aftermath of critical illness. The suddenness and intensity of ICU admission, combined with invasive treatments and life support measures present in an unfamiliar setting, can be deeply traumatic. Disturbing hallucinations, delirium, or the stark remembrance of near-death instances can persist, leading to chronic avoidance behaviors, recurrent nightmares, or hyper-alertness well beyond physical recovery. PTSD's strain can obstruct rehabilitation, impair relationships, and erode overall life satisfaction [7]. The high prevalence of PTSD symptoms in ICU survivors emphasizes the need for a comprehensive post-discharge regimen that integrates physical recovery with proactive psychological oversight.

Current research highlights the manifestation of post-traumatic symptoms following ICU-associated traumatic experiences, indicating a subsequent decline in life quality, marked by elevated anxiety, stress, and depression [4]. A previous study illustrated this trend, revealing notable percentages of anxiety, depression, and PTSD post-ICU admission. Moreover, a tangible correlation was discerned between anxiety symptoms and the duration of ICU tenure [8]. Another UK study reiterated these findings, underscoring the nexus between anxiety, depression, and PTSD in ICU survivors and its potential effect on their long-term prognosis [2].

Recognizing that former ICU survivors are more susceptible to psychiatric disorders than other hospital inpatients, this study investigates the prevalence and correlations of depression, anxiety, and PTSD among ICU survivors in Saudi Arabia's Jazan region. Given the significant lack of regional data, our research fills a crucial gap, offering essential insights that can inform tailored healthcare interventions and influence mental health policies for this area.

## Materials and methods

Study design and population

This investigation utilized a hospital-based cross-sectional study, focusing on the acquisition of descriptive quantitative metrics to evaluate the prevalence and contributory factors related to depression, anxiety, and PTSD among survivors of ICUs in the Jazan Region of Saudi Arabia. The research was undertaken in two primary healthcare settings: Prince Mohammed Bin Nasser Hospital and King Fahad Central Hospital. These facilities are situated in the southwestern region of Saudi Arabia. Inclusion criteria were delineated to encompass individuals who were at least 18 years of age, had more than five days of ICU admission history, expressed willingness to participate in the study, and were residents of the Jazan Region. Using G*Power software, we determined the necessary sample size for our study based on a large anticipated effect size (Cohen's d of 0.8), a high power of 0.95 to minimize Type II errors, and a standard alpha of 0.05. The analysis indicated that a minimum of 88 participants is required to ensure the robustness of t-test analyses for detecting significant differences in psychological symptoms among post-ICU patients. We employed a convenience sampling method to select a diverse sample of 100 participants. Patients were approached by trained data collectors who introduced the study objectives and obtained informed consent during the initial screening process in the hospital setting. Participants were recruited during their follow-up visits one week after ICU discharge. The face-to-face interviews were conducted as part of the data collection process. Data collection took place over six months, from September 2023 to February 2024.

Ethical considerations

Ethical clearance was obtained from the Jazan Health Ethics Committee (Approval No. 2319). A written informed consent was mandatorily acquired from all participants, who retained the freedom to withdraw from the study at any stage without repercussions. Stringent confidentiality and privacy protocols were observed.

Data collection tool

Data variables included demographic factors such as age, gender, educational level, marital status, occupation, monthly income, and duration of ICU stay. Psychometric instruments employed were the hospital anxiety and depression scale (HADS) and the post-trauma symptom scale (PTSS-10) to quantify symptoms of anxiety, depression, and trauma response. The HADS is a psychometric instrument comprising two distinct subscales designed to assess symptoms of anxiety and depression. Each subscale incorporates seven items, rated on a frequency four-point Likert scale (rated from 3 to 0), describing how they felt in the past week. The cumulative scores for each subscale can range from 0 to 21. Interpretation of the scores is categorized into three levels of severity: low/normal (0-7), moderate/borderline abnormal (8-14), and high/ abnormal (15-21), allowing for a nuanced assessment of the psychological conditions under investigation.

We utilized the PTSS-10, a robust self-assessment tool consisting of 10 items, to measure the occurrence and severity of PTSD symptoms among participants. This instrument was selected due to its proven reliability and validity in diverse populations, as established by Mehlum and Weisaeth [9]. It enables a nuanced understanding of PTSD symptoms in ICU survivors, facilitating precise assessments crucial for effective post-discharge care planning. Each symptom is evaluated using a 7-point Likert scale, where 1 denotes "not at all/never", and 7 denotes "very often" [9]. The symptoms encompass sleep disturbances, nightmares, depressive feelings, heightened startle responses, tendencies to isolate, irritability, emotional fluctuations, guilt or diminished self-worth, apprehension of triggers reminiscent of the traumatic incident, and physical tension. The PTSS-10 is acknowledged for its strong internal coherence and consistency over repeated tests and has been recognized as an effective, valid, and dependable tool for PTSD screening [10,11]. Scores can range from 10 to 70, with a score exceeding 34 indicating a potential PTSD diagnosis [11].

Both instruments have undergone validity and reliability testing in previous research endeavors, substantiating their applicability in diverse clinical settings [10,11]. Moreover, the Arabic-validated version [12] was used to align with the linguistic and cultural context of the Jazan Region, further enhancing their contextual relevance and facilitating more accurate assessments.

Data analysis

After collecting the data, we utilized Microsoft Excel for initial coding and data organization. The statistical analyses were conducted using the Statistical Package for the Social Sciences (SPSS) software, Version 26. Categorical variables were represented as counts and percentages, while continuous variables were initially depicted as mean ± standard deviation. Prior to applying parametric tests, we assessed the normality of the distribution of continuous variables using the Shapiro-Wilk test. Based on these results, where data did not follow a normal distribution, non-parametric tests were chosen. Thus, for non-normally distributed continuous variables, we employed the Mann-Whitney test for comparisons between two groups and the Kruskal-Wallis test, a non-parametric alternative to one-way ANOVA, for comparisons across more than two groups. Relationships between scores were explored using the Spearman correlation coefficient due to the non-normal distribution of our data. A p-value of less than 0.05 was set as the criterion for statistical significance.

## Results

In a cohort of 100 ICU survivors from the Jazan Region, the predominant age group was 36-60 years (49%), with an almost equal gender distribution of 53% male and 47% female. Most participants (70%) had secondary education or less, and the majority were married (63%). The employment status showed that 52% were unemployed, while monthly income figures indicated that 44% earned less than 3000 Saudi Riyals (SAR). The majority of the sample had a short ICU stay, with 87% staying less than seven days.

Significant associations were observed between demographic factors and psychological distress scores (Table 1). Gender significantly influenced anxiety scores (p=0.005), with females having a higher mean (8.26±4.34) than males (5.7±4.69). Education level showed a significant association with depression scores (p=0.003), where individuals with secondary education or less had a higher mean score (8.46±3.14) compared to those with a bachelor's or higher (6.43±2.3). Unemployed participants registered higher depression scores (8.77±3.05) than employed ones (6.51±2.48, p=0.004). Monthly income was also significant; those earning between 5000-10,000 SAR showed the highest mean anxiety score (10.18±4.92, p=0.012), and those earning less than 3000 SAR had lower PTSS scores (11.48±11.85, p=0.022). Finally, marital status was significantly associated with depression scores (p=0.021), with married individuals exhibiting higher scores (8.37±2.98) than those single or separated (6.97±3).

**Table 1:** Demographic and clinical characteristics of ICU survivors in Jazan SAR: Saudi Riyal

Characteristic	N (%)	Anxiety score		Depression score		Post-trauma symptom scale score	
		Mean (SD)	p-value	Mean (SD)	p-value	Mean (SD)	p-value
Gender							
Male	53 (53%)	5.7 (4.69)	0.005	8.04 (2.83)	0.289	15.09 (12.81)	0.939
Female	47 (47%)	8.26 (4.34)		7.64 (3.29)		14.79 (11.15)	
Age groups							
< 35 years	37 (37%)	6.78 (4.22)	0.866	6.81 (2.59)	0.021	14.57 (14.26)	0.624
36-60 years	49 (49%)	7.16 (5.16)		8.12 (2.92)		15.16 (11.31)	
> 60 years	14 (14%)	6.29 (4.34)		9.64 (3.71)		15.21 (7.77)	
Education level							
Secondary or less	70 (70%)	6.41 (4.67)	0.081	8.46 (3.14)	0.003	14.79 (12.21)	0.709
Bachelor's or higher	30 (30%)	8.03 (4.6)		6.43 (2.3)		15.33 (11.67)	
Marital Status							
Single/Separated	37 (37%)	6.73 (4.85)	0.686	6.97 (3)	0.021	14.03 (12.68)	0.411
Married	63 (63%)	7 (4.62)		8.37 (2.98)		15.49 (11.65)	
Work status							
Unemployed	52 (52%)	6.63 (4.22)	0.906	8.77 (3.05)	0.004	13.94 (12.15)	0.352
Employed	39 (39%)	7.23 (5.38)		6.51 (2.48)		15.49 (12.39)	
Retired	9 (9.0%)	7 (4.44)		8.33 (3.43)		18.44 (9.41)	
Monthly income SAR							
Less than 3000	44 (44%)	6.2 (4.59)	0.012	8.59 (3.24)	0.058	11.48 (11.85)	0.022
Between 3000-5000	26 (26%)	5.38 (3.58)		8.04 (3.04)		16.19 (9.61)	
Between 5000 - 10000	11 (11%)	10.18 (4.92)		6.55 (2.25)		21.64 (13.39)	
More than 10,000	19 (19%)	8.68 (4.98)		6.63 (2.5)		17.42 (12.76)	
Length of stay in the ICU							
< 7 days	87 (87%)	6.55 (4.74)	0.087	7.86 (2.97)	0.183	14.68 (12.43)	0.305
7 - 14 days	8 (8.0%)	8.88 (3.56)		6.5 (2.78)		14 (8.98)	
> 14 days	5 (5.0%)	9.8 (4.21)		9.8 (4.32)		21.2 (6.83)	

**Figure 1:**
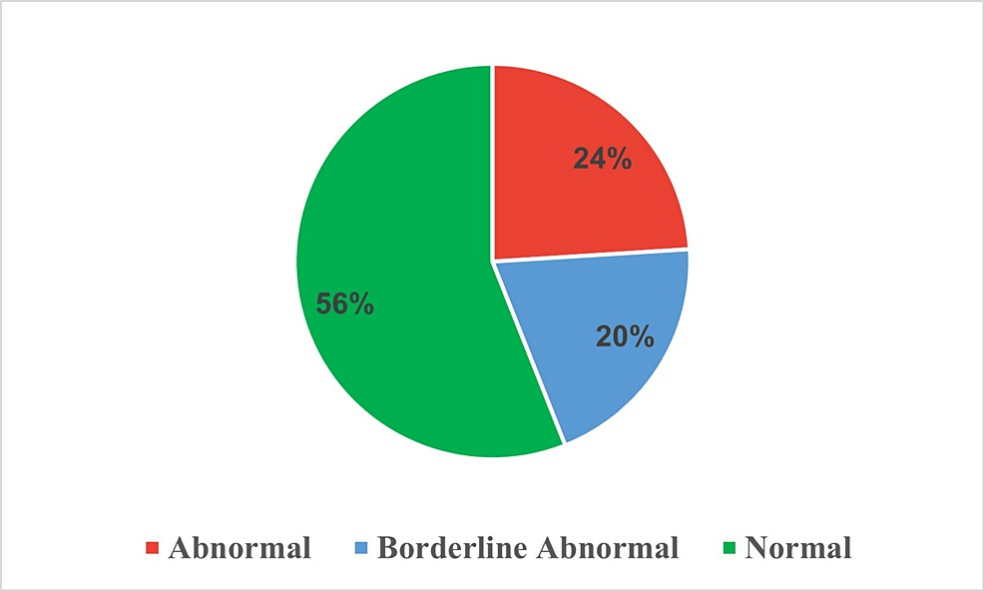
Classification of anxiety severity levels among intensive care unit survivors.

The ICU survivors' responses to seven anxiety-related questions using a 0-to-3 scale (Table 2). For feeling tense, 56.0% didn't feel it, while 4.0% did frequently. Concerning frightful anticipations, 42.0% didn't experience it, and 8.0% felt it strongly. On worrying thoughts, 35.0% occasionally worried, and 5.0% worried a lot. For sitting at ease, 24.0% could, while an equal percentage couldn't. Regarding frightened feelings like 'butterflies' in the stomach, 41.0% never felt them, and 11.0% did often. With restlessness, 43.0% weren't restless, but 6.0% were. For sudden panic, 47.0% hadn't felt it, versus 9.0% who often did. Moreover, when the data was aggregated into categories of anxiety severity, a majority of participants (56.0%) fell into the 'normal' range, 24.0% were classified as 'abnormal,' and 20.0% were in the 'borderline abnormal' category (Figure 1).

**Table 2:** Frequency distribution of responses to anxiety-related questions among intensive care unit survivors.

Anxiety items	0	1	2	3
I feel tense or 'wound up'	56 (56.0%)	28 (28.0%)	12 (12.0%)	4 (4.0%)
I get a sort of frightened feeling as if something awful is about to happen	42 (42.0%)	30 (30.0%)	20 (20.0%)	8 (8.0%)
Worrying thoughts go through my mind	35 (35.0%)	39 (39.0%)	21 (21.0%)	5 (5.0%)
I can sit at ease and feel relaxed	24 (24.0%)	18 (18.0%)	34 (34.0%)	24 (24.0%)
I get a sort of frightened feeling like 'butterflies' in the stomach	41 (41.0%)	36 (36.0%)	12 (12.0%)	11 (11.0%)
I feel restless as I have to be on the move	43 (43.0%)	31 (31.0%)	20 (20.0%)	6 (6.0%)
I get sudden feelings of panic	47 (47.0%)	19 (19.0%)	25 (25.0%)	9 (9.0%)

**Figure 2:**
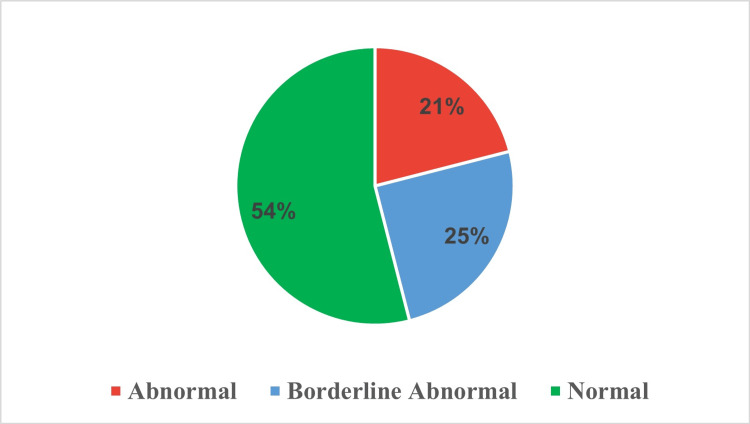
Classification of depression severity levels among intensive care unit survivors.

The responses of ICU survivors to seven depression-related questions using a 0-to-3 severity scale (Table 3). Regarding enjoyment of previous activities, 38.0% noted no change, while 16.0% hardly enjoyed them. On humor perception, 55.0% responded positively, while 10.0% couldn't laugh at all. Only 21.0% felt cheerful often, compared to 17.0% who rarely did. While 52.0% didn't feel slowed down, 5.0% often did. Concerning self-care, 54.0% admitted negligence. On anticipation, 45.0% were as enthusiastic as before, but 24.0% rarely looked forward. Finally, for media enjoyment, 18.0% enjoyed it often, whereas 20.0% seldom did. Upon evaluating the aggregated scores, the overall distribution of depression symptoms among ICU survivors is as follows: 54% are categorized as 'Normal,' 25% as 'borderline abnormal,' and 21% as 'abnormal' (Figure 2).

**Table 3:** Frequency distribution of responses to depression-related questions among intensive care unit survivors.

Depression items	0	1	2	3
I still enjoy the things I used to enjoy	38 (38.0%)	38 (38.0%)	8 (8.0%)	16 (16.0%)
I can laugh and see the funny side of things	55 (55.0%)	29 (29.0%)	6 (6.0%)	10 (10.0%)
I feel cheerful	21 (21.0%)	38 (38.0%)	24 (24.0%)	17 (17.0%)
I feel as if I am slowed down	52 (52.0%)	30 (30.0%)	13 (13.0%)	5 (5.0%)
I have lost interest in my appearance	32 (32.0%)	9 (9.0%)	54 (54.0%)	5 (5.0%)
I look forward with enjoyment to things	45 (45.0%)	18 (18.0%)	13 (13.0%)	24 (24.0%)
I can enjoy a good book or radio or TV program	18 (18.0%)	28 (28.0%)	34 (34.0%)	20 (20.0%)

**Figure 3:**
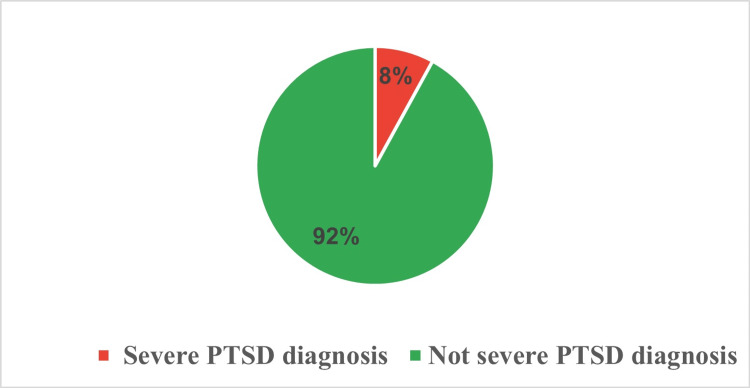
Distribution of post-traumatic stress disorder severity among intensive care unit survivors. PTSD: Post-traumatic stress disorder

The frequency distribution of specific PTSD symptoms among ICU survivors (Table 4). Mood swings emerged as the most prevalent symptom, registering an average score of 2.26, although 41.0% of participants were unaffected. Following closely, irritability had an average score of 2.15, with 45.0% of the participants reporting no such feelings. Sleep problems came next with an average score of 2.17; while 38.0% of participants did not face any issues. In contrast, symptoms like fear of recollection and bad conscience are less frequently reported. Most symptoms, such as depression and jumpiness, show a majority of respondents clustered at the lower severity end, although a non-negligible minority experience these symptoms at higher severity levels. Figure 3 provides a view of PTSD diagnosis among ICU survivors. While 92% are categorized under 'not severe PTSD diagnosis,' a significant 8% are diagnosed with 'severe PTSD' (Figure 3). This prevalence underscores the importance of mental health evaluation and targeted interventions for this vulnerable population.

**Table 4:** Rates of the items of PTSS among intensive care unit survivors PTSS: Post-trauma symptom scale

PTSS items	0	1	2	3	4	5	6	Average
Sleep problems	38 (38.0%)	6 (6.0%)	11 (11.0%)	16 (16.0%)	12 (12.0%)	9 (9.0%)	8 (8.0%)	2.17
Nightmares	57 (57.0%)	9 (9.0%)	8 (8.0%)	8 (8.0%)	11 (11.0%)	2 (2.0%)	5 (5.0%)	1.33
Depression	56 (56.0%)	8 (8.0%)	11 (11.0%)	12 (12.0%)	6 (6.0%)	3 (3.0%)	4 (4.0%)	1.29
Jumpiness	51 (51.0%)	5 (5.0%)	11 (11.0%)	10 (10.0%)	10 (10.0%)	6 (6.0%)	7 (7.0%)	1.69
Need to withdraw	59 (59.0%)	5 (5.0%)	6 (6.0%)	10 (10.0%)	4 (4.0%)	4 (4.0%)	12 (12.0%)	1.55
Irritability	45 (45.0%)	2 (2.0%)	7 (7.0%)	16 (16.0%)	11 (11.0%)	7 (7.0%)	12 (12.0%)	2.15
Mood swings	41 (41.0%)	4 (4.0%)	6 (6.0%)	14 (14.0%)	16 (16.0%)	10 (10.0%)	9 (9.0%)	2.26
Bad conscience	72 (72.0%)	3 (3.0%)	7 (7.0%)	5 (5.0%)	3 (3.0%)	7 (7.0%)	3 (3.0%)	0.97
Fear of recollections	79 (79.0%)	3 (3.0%)	5 (5.0%)	5 (5.0%)	4 (4.0%)	1 (1.0%)	3 (3.0%)	0.67
Muscular tensions	75 (75.0%)	4 (4.0%)	6 (6.0%)	4 (4.0%)	3 (3.0%)	1 (1.0%)	7 (7.0%)	0.87

The anxiety scores were characterized by a mean of 6.9±4.69 (range 0-18). Depression scores had a mean of 7.85±3.05 (range 3-15). PTSS scores demonstrated the highest variability, with a mean of 14.95±12 (range 0-49). The correlation matrix among these three variables (Table 5). Anxiety and depression scores had a low, non-significant Spearman's rho correlation coefficient of 0.08 (p=0.406), suggesting little relationship between the two. Conversely, anxiety and PTSS scores were moderately correlated with a Spearman's rho of 0.38, which was statistically significant (p=0.001). Depression and PTSS had a weaker, albeit positive, Spearman's rho of 0.18, but this was not statistically significant (p=0.071). These correlations indicate that anxiety is more closely related to PTSS than to depression in this sample of ICU survivors.

**Table 5:** Correlation matrix of the mean scores of anxiety, depression, and post-trauma symptom scale

Variable		Anxiety	Depression	Post Trauma Symptom Scale
Anxiety	Spearman's rho	—		
	p-value	—		
Depression	Spearman's rho	0.08	—	
	p-value	0.406	—	
Post-trauma Symptom Scale	Spearman's rho	0.38	0.18	—
	p-value		0.071	—

## Discussion

Our study provides an analysis of the prevalence and correlations of depression, anxiety, and PTSD among ICU survivors in Saudi Arabia's Jazan region. This investigation is crucial due to the notable absence of region-specific data, which is essential for developing targeted mental health strategies and interventions tailored to the unique needs of this population.

The majority of ICU survivors exhibit 'normal' levels of anxiety and depression. however, a significant proportion falls under the 'borderline abnormal' or 'abnormal' category. Such observations accentuate the imperative for comprehensive mental health evaluation for ICU survivors. Targeted interventions could greatly benefit this subset. Studies consistently indicate that a significant fraction of ICU survivors grapple with anxiety and depression symptoms leaning towards the "borderline abnormal" or "abnormal" spectrum. Specifically, a recent study observed that 22% showed anxiety symptoms, while 36% displayed depressive symptoms [13]. Meanwhile, a study reported prevalence rates of 60.3% for anxiety and 26.4% for depression [8]. Furthermore, Wang et al. elucidated that ICU survivors with pronounced psychiatric comorbidities, encompassing anxiety, depression, and PTSD, generally have a diminished quality of life [14].

Many ICU survivors experience a range of post-care symptoms, with sleep disturbances being particularly pronounced, suggesting the need for targeted post-ICU sleep interventions. Although the majority escape a severe PTSD diagnosis, an 8% prevalence rate for 'Severe PTSD' remains alarming. Research consistently underscores prevalent issues among ICU survivors, such as anxiety, depression, feelings of isolation, and fear. Several studies delineate the negative experiences of ICU survivors, identifying symptoms like sleep deprivation, anxiety, isolation, fatigue, and pain [15-17]. Concerning PTSD, A systematic review and meta-analysis found the overall prevalence of PTSD symptoms in adult critical care survivors to be 19.83% [18]. Nevertheless, Davydow calls attention to the imperative of further studies on post-ICU PTSD risk factors and interventions [19]. Griffiths et al. also stressed the need for a unified stance on PTSD's true prevalence and the best assessment methods for ICU survivors [20].

Regarding the correlation between anxiety, depression, and PTSS, it is noteworthy that while anxiety and depression showed a negligible correlation, a moderate, significant relationship was observed between anxiety and PTSS. This suggests that in ICU survivors, anxiety may be an early indicator of potential PTSS, reinforcing the need for early screening and intervention in ICU survivors. A previous study found that symptoms of post-traumatic stress, anxiety, and depression assessed 1 week after ICU stay correlated with psychological outcomes [21].

The results of our study reveal significant psychological distress among ICU survivors, with notable variance across different demographic groups. A significant association was observed between gender and anxiety, with females displaying higher scores, emphasizing the gender disparity in the psychological aftermath of ICU experiences. The influence of socioeconomic factors on psychological outcomes was also evident. Participants with secondary education or less exhibited higher depression scores, and unemployment was linked to elevated depression scores, suggesting that education and employment might act as protective factors against post-ICU distress. Household income exhibited a significant association with PTSS scores. The association of marital status with depression underlines the potential psychological implications of relational status in ICU survivors. Among ICU survivors, female gender is linked to heightened anxiety and worse psychological recovery, as supported by previous studies [22-24]. Socioeconomic factors, particularly income, are associated with increased anxiety, PTSD, and depression [8,25,26]. Additionally, factors like age, education level, marital and employment status influence depression in ICU survivors [14,23].

Sociodemographic factors play a significant role in shaping individual experiences of trauma and recovery trajectories, interacting with established theoretical models. Herman's Three Stage Model emphasizes the importance of establishing safety, remembrance and mourning, and reconnection, all of which are influenced by socioeconomic status, cultural norms, education, and social support. Similarly, the Post-traumatic Growth (PTG) Model highlights how age, education, and pre-existing social networks affect personal strength, openness to new possibilities, relational improvements, life appreciation, and spiritual change post-trauma [27]. The Biopsychosocial Model for PTSD Recovery underscores the role of access to healthcare, psychological understanding, and community support in mediating recovery, which are all shaped by sociodemographic variables [28]. Likewise, the Empowerment Psychological Intervention Model emphasizes that language, education level, and access to resources impact trauma education and the development of coping skills, while socioeconomic and cultural positioning influence the level of support and the ability to actively participate in treatment planning. This integrated perspective reveals that trauma recovery is not merely a psychological journey but is deeply embedded in the economic, cultural, and social fabric of an individual's environment. Consequently, effective trauma recovery strategies necessitate tailored interventions that are culturally sensitive and socioeconomically accessible to enhance their effectiveness. By recognizing the complex interplay between sociodemographic factors and trauma recovery, mental health professionals can develop more comprehensive and inclusive approaches to support individuals in their healing process [27,28].

Strengths and limitations

This study leverages the use of validated instruments, ensuring the reliability and accuracy of the data collected on the psychological impacts experienced by ICU survivors. Additionally, the demographic diversity of the sample enhances the relevance and applicability of our findings across different subgroups within the ICU survivor population. However, the small sample size, use of convenience sampling, and a cross-sectional design collectively restrict the generalizability of the results and prevent causal inferences. Additionally, the study's limited regional focus further constrains the broader applicability of the findings. It is important to clarify that identified associations do not imply causality, and more extensive longitudinal studies are needed to validate and expand upon these findings, offering a clearer understanding of long-term psychological impacts in this population.

## Conclusions

The study underlines the multifaceted psychological repercussions experienced by ICU survivors. Among the participants, 24% were classified as 'abnormal' and 20% as 'borderline abnormal' for anxiety, while 25% were 'borderline abnormal' and 21% 'abnormal' for depression. About 8% of participants were diagnosed with severe PTSD. Anxiety was more strongly correlated with post-traumatic stress symptoms than depression. The study found significant associations between distress scores and factors such as gender, education, employment status, income, and marital status. These results highlight the need for comprehensive mental health evaluations and tailored interventions to support the psychological well-being of ICU survivors.
